# Inter‐ and Intraspecific Variation in Web Architecture and Its Significance in Australian Funnel‐Web Spiders (Atracidae)

**DOI:** 10.1002/ece3.74473

**Published:** 2026-10-14

**Authors:** Noah Brown, Thomas E. White

**Affiliations:** ^1^ School of Animal and Veterinary Sciences The University of Sydney Sydney New South Wales Australia; ^2^ School of Life and Environmental Science The University of Sydney Sydney New South Wales Australia

**Keywords:** Mygalomorphae, silk use, structural diversity, web design, web function

## Abstract

Web building is a defining trait of spiders, encompassing a remarkable diversity of architectures and functions. However, the order Mygalomorphae remains underrepresented compared to Araneomorphae, with limited research on their structural diversity and function. Mygalomorph designs provide valuable insights into the evolution of web‐building and silk use in spiders. In this study, we described and quantified inter‐ and intraspecific variation in web architecture across three Atracidae species: *Hadronyche cerberea* , *H. versuta* and the *Atrax robustus* complex. Our research represents the first quantitative characterisation of web architecture within Atracidae. Using image‐based morphometric analysis, we quantified structural traits that statistically differentiated web designs among species. In addition, we refined previous descriptions of their web structures through detailed qualitative accounts and schematic diagrams to enhance species identification and understanding of web architecture. Collectively, our findings revealed clear qualitative and quantitative differences among species, with several structural traits responsible for these variations. These results provide a foundation for exploring the ecological drivers and functional significance of web diversity in Atracidae, contributing to a broader understanding of mygalomorph evolution and behaviour.

## Graphical abstract

We quantified inter‐ and intraspecific variation in web architecture across three Australian funnel‐web spider species using image‐based morphometrics. Species differed significantly in web size, shape and sensory‐to‐capture zone allocation and these differences support both ecological interpretation and non‐invasive field identification.

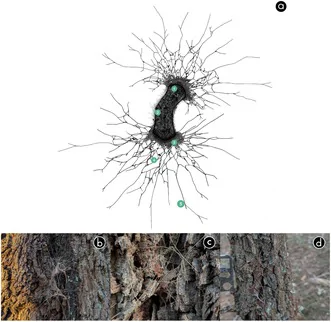

## 1 Introduction

Web construction is an ancestral trait in spiders (Araneae), with these webs varying remarkably in architecture and function. Silk fulfils multiple essential roles, including reproduction, egg sac construction, prey capture, traversal, predator protection and environmental buffering (Vollrath and Selden 2007). The structures that serve these roles vary widely across taxa, offering insight into the ecological and adaptive pressures shaping web evolution. In particular, web architecture provides a functional interface between the organism and its environment, mediating trade‐offs between prey detection, capture efficiency and protection.

Most detailed work on web diversity and function has focused on Araneomorphae, especially aerial web builders such as orb weavers, sheet weavers and tangle‐web spiders, which display the greatest structural diversity and the archetypal web forms associated with spiders (Blackledge et al. 2009; Coddington et al. 2019; Eberhard 2020). In contrast, the infraorder Mygalomorphae, which includes tarantulas, trapdoor spiders, funnel‐web spiders and mouse spiders, has received comparatively limited attention with respect to the description, variation and functional interpretation of silk architectures (Coddington et al. 2019; Coyle 1986; Opatova et al. 2019; Wilson et al. 2023). Although mygalomorphs are generally more sedentary and exhibit less overt architectural diversity than araneomorphs, they construct a range of silk structures, most commonly associated with burrow entrances and surrounding retreats (Blackledge et al. 2009; Eberhard 2020).

Functionally, these structures are tightly linked to the sensory ecology of mygalomorphs. In keeping with most spiders, mygalomorphs are not strongly visual and instead detect and localise prey primarily through substrate‐borne vibrations transmitted through silk and surrounding materials (Buchli 1969; Den Otter 1973). As a result, web and burrow architectures are often shaped by trade‐offs between extending sensory and foraging range and maintaining protection from predators and environmental exposure (Coyle 1986). For example, extended sheet or funnel‐like structures can increase sensory coverage and interception opportunities but may reduce concealment, whereas trapdoors and heavily reinforced retreats prioritise protection at the expense of foraging range. Many species appear to construct intermediate designs that mitigate these trade‐offs, such as incorporating sense‐lines or dense retreats that simultaneously enhance sensory detection and defence (Bond and Coyle 1995; Coyle 1981, 1986; Wilson et al. 2023). Despite this general functional framework, the specific structural variation of mygalomorph webs and how this variation relates to prey capture and sensory function, remains poorly quantified.

The family Atracidae (Australian funnel‐web spiders) provides a particularly suitable system for investigating these questions. Comprising 39 species across the genera *Atrax*, *Hadronyche* and *Illawarra*, atracids typically occupy moist, sheltered microhabitats along the east coast of Australia and spend the majority of their lives within burrow retreats (Gray 2010; Loria et al. 2025; Raven and Douglas 2025). They are opportunistic web builders, constructing funnel‐web entrances in crevices, embankments, logs and tree cavities, where silk structures are thought to contribute to both prey detection and capture (Gray 2010; Wilson et al. 2023). Previous descriptions suggest genus‐level differences in web form, with Atrax primarily building entrance‐associated structures and *Hadronyche* exhibiting a wider range of architectures, including open‐hole, trapdoor‐like and lattice‐based designs (Gray 2010; Wilson et al. 2023). However, most existing accounts are brief taxonomic or natural history descriptions and systematic, quantitative analyses of atracid web architecture are lacking. Consequently, the extent of inter‐ and intraspecific variation, as well as its potential ecological significance, is unresolved.

Our three focal taxa co‐occur across coastal and montane New South Wales, span the major architectural categories described in prior accounts (entrance‐associated, lattice and open‐hole forms; Gray 2010) and are the species most frequently encountered during medical and antivenom sampling in the region; together they offer a tractable comparative system that captures much of the recorded structural diversity in the family. Here, we quantify inter‐ and intraspecific variation in the silk architectures of three atracid spiders— *Hadronyche cerberea* , *Hadronyche versuta* and the *Atrax robustus* complex—using field‐based observations, image‐based morphometrics and qualitative web descriptions supported by schematic representations. By measuring key architectural metrics and explicitly distinguishing sensory and capture zones, we aim to (1) characterise structural variation in atracid webs, (2) identify the principal axes of morphological differentiation among species and web regions and (3) provide a quantitative foundation for interpreting the ecological and behavioural significance of web architecture in this understudied lineage.

## 2 Methods

### 2.1 Field‐Based Web Sampling

We sampled funnel‐web burrows across eight sites in three regions of New South Wales: the Central Coast, Greater Sydney (including the Blue Mountains, Upper North Shore and Macarthur) and the Northern Southern Highlands (Figure S1). We chose sites within the known geographic ranges of the *Atrax robustus* complex, *Hadronyche cerberea* and *Hadronyche versuta* (Gray 2010; Loria et al. 2025).

We searched opportunistically but systematically through microhabitats likely to contain Atracidae, including embankments, logs, crevices and ground vegetation. When we located a web, we photographed it with a Google Pixel 7, positioning the camera as close to perpendicular to the web surface as possible so we captured the full structure. When necessary, we took photographs from multiple distances to include the full extent of radiating triplines. We placed a scale reference within each image for calibration. We removed vegetation or loose debris only when it obscured the web and only when we could do so without altering web structure.

### 2.2 Species Assignment and Image Screening

We recorded three taxa across all sites: *Hadronyche cerberea* , *Hadronyche versuta* and members of the *Atrax robustus* complex. Because *Atrax robustus* has recently been split into *A. montanus* , *A. robustus* and *A. christenseni* , we could not reliably identify web samples beyond the complex level in the field (Loria et al. 2025). We observed no consistent structural differences among these taxa during sampling and published descriptions are limited, so we treated all individuals as the *Atrax robustus* complex for analysis to remain consistent with prior literature. We did not encounter *Hadronyche mascordi* or *H. levittgreggae*. Identification was based on a combination of web and burrow architecture and external morphology of spiders visible at the burrow entrance at the time of photography (carapace and abdominal colouration and patterning, leg banding and tube vs. open‐hole entrance form), evaluated against the regional taxonomic keys of Gray (2010). Consistent with the non‐invasive intent of the study and the medical risk associated with Atracidae, we did not collect, handle or excavate individuals. We therefore did not assign sex or developmental stage for most spiders, although web size, entrance dimensions and the presence of clustered juvenile burrows (see Results) indicate that our samples spanned both juveniles and adults of both sexes.

Before analysis, we screened images and excluded those with substantial environmental obscurity, inadequate focus or incomplete web structures. Our final sample sizes were *Atrax robustus* complex (*n* = 13), *Hadronyche cerberea* (*n* = 13) and *Hadronyche versuta* (*n* = 9).

### 2.3 Image Calibration and Analysis

We imported all retained images into Fiji (ImageJ 2.16/0.154p) (Schindelin et al. 2012) and calibrated each image (mm per pixel) using the in‐frame scale reference. For each web, we delineated two regions of interest (Figure 1). By way of brief background, atracids are sit‐and‐wait ambush predators that remain within the burrow until prey contact a tripline or the silk near the entrance, at which point the spider rushes from the retreat to seize the prey with its chelicerae and drag it back into the burrow (Gray 2010). The structural division between extended traplines and denser entrance‐associated silk thus maps directly onto the detection and interception phases of capture. We defined the sensory zone (SZ) as the full extent of the web, including all radiating triplines, representing the area over which the spider could plausibly detect prey vibrations. We defined the capture zone (CZ) as the subset of the web near the burrow entrance that showed visibly denser silk threads, corresponding to the region most likely involved in prey interception.

**FIGURE 1:**
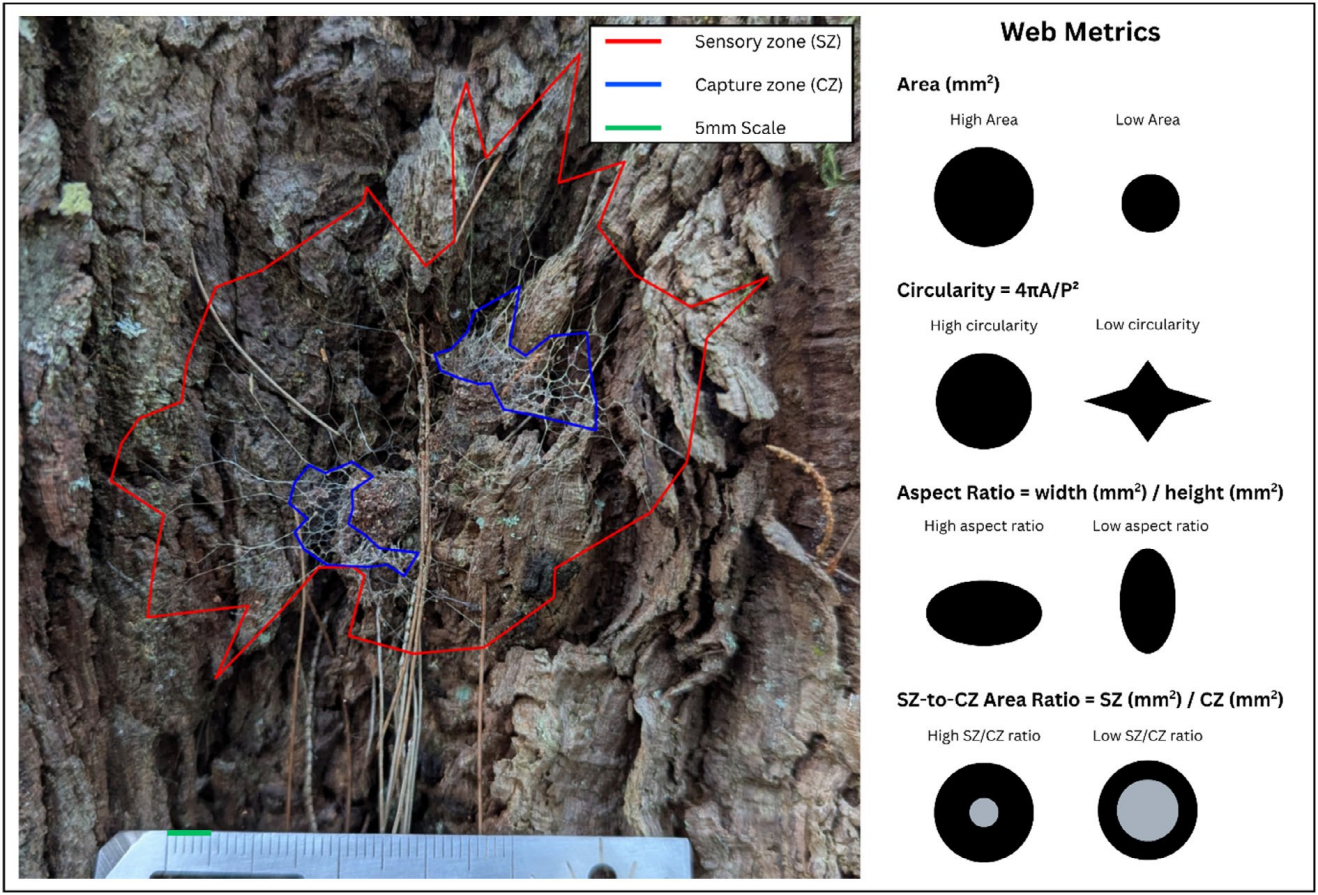
Diagram outlining the methodology. Left: Image processing workflow showing differentiation of capture and sensory zones and determination of scale. Right: Web metrics extracted from the processed data for quantitative analysis, with representative model examples provided for each metric.

### 2.4 Qualitative Analysis

In the field, we recorded qualitative observations alongside photographs, including burrow structure, entrance orientation, number of entrances, surrounding vegetation type (e.g., dry vs. wet sclerophyll forest, dominant tree species in the immediate vicinity of the burrow) and substrate type. These habitat data were qualitative descriptors used for context only and were not quantified or included in the formal analyses below and so we report them in the species‐level web descriptions in the Results. We used these observations to refine species‐level web descriptions, develop schematic representations of typical architectures and contextualise the quantitative analyses.

### 2.5 Quantitative Analysis

We analysed all data in R (v[4.5.1]; R Core Team 2026; Posit team 2025) using measurements extracted from calibrated images. For each web, we manually traced the boundaries of the sensory and capture zones to obtain coordinate sets for each region and then generated convex hull polygons to approximate the spatial extent of each zone. For the SZ, the boundary connected the outermost terminations of the radiating triplines, while for the CZ the boundary followed the visible outer margin of the dense silk lattice or sheet surrounding the entrance(s). From these polygons, we extracted four morphometric metrics (Figure 1): area (mm^2^), circularity (4π × area/perimeter^2^), aspect ratio (maximum width/maximum height) and sensory‐to‐capture allocation (SZ area/CZ area). We selected these metrics to capture overall web size, shape and relative allocation between broader sensory coverage and denser capture structure. More specifically, web area indexes overall investment in silk and the spatial scale at which sensory and capture functions operate; circularity captures the regularity of the web outline, distinguishing roughly enclosed designs from elongate or asymmetric architectures and providing a simple, scale‐free descriptor that complements area; width and height refer to the maximum horizontal and vertical extents of the convex hull, respectively and aspect ratio (width/height) quantifies the dominant axis of extension, which is informative because mygalomorph webs are often oriented along substrate features (e.g., tree trunks, embankments) that may channel prey movement; and the SZ:CZ ratio summarises functional allocation between detection (extended triplines) and interception (denser silk near the entrance). We note two limitations of these descriptors. First, our SZ convex hull treats the area enclosed by the outermost triplines as a single sensory polygon, even though prey passing between adjacent triplines may not necessarily be detected; SZ values are therefore best interpreted as a relative measure of sensory coverage rather than an absolute estimate of detection area. Second, all metrics are influenced by the local microhabitat in which a web is built and, presumably, by spider body size; we partly account for these by reporting both absolute and shape‐based descriptors and by treating intraspecific variation as a quantity of interest in its own right. Note that all values below are reported as mean ± standard deviation.

We used the morphometric variables for both descriptive summaries and multivariate inference. We ran a principal component analysis (PCA) implemented using the prcomp function in base R to summarise the main axes of variation in web architecture across species and web regions. Prior to analysis, web area and SZ:CZ ratio were log‐transformed (natural log); all four variables were then scaled to unit variance and mean‐centred. We then used permutational multivariate analysis of variance (PERMANOVA) (adonis2 function, vegan package [v2.7‐3; Oksanen et al. 2024]) on Euclidean distance matrices with 9999 permutations to test for differences in overall web morphology among species and between web zones, including the main effects of species and zone, using a multivariate framework that accommodates correlation among traits. Pairwise PERMANOVA was conducted for each species pair using separate adonis2 calls, with Holm correction applied to raw *p* values across the three comparisons.

## 3 Results

### 3.1 Web Descriptions

Species accounts below are ordered by web architecture rather than by taxonomy, following the sequence of entrance‐associated, lattice and open‐hole forms introduced above (Gray 2010).

#### 3.1.1 Hadronyche cerberea

*Hadronyche cerberea* is distributed along the south‐eastern coast of New South Wales and all individuals we recorded occurred on the Central Coast, near the northern extent of the species' range. We found this species exclusively in arboreal contexts, typically in open forested habitats (dry and wet sclerophyll) and most commonly associated with *Casuarina* and other tree species such as *Melaleuca*, *Banksia* and *Eucalyptus* (Gray 2010). By ‘opportunistically’ we mean that, rather than excavating de novo, individuals occupied and silk‐lined whatever pre‐existing cavities were available within the local substrate. Specifically, individuals constructed burrows within fissures, heartwood pipes, borer holes and rotted sections of trunks and branches. Burrow height varied but was largely constrained by the availability of suitable cavities rather than a fixed vertical preference. We frequently observed multiple individuals within the same tree, suggesting local clustering at suitable substrates and/or natal philopatry.

The stereotypical web design we recorded featured an entrance characterised by a convex, crescent‐shaped, silk‐sock‐like flap that lies flush against the web when not in use (Figure 2a). Webs typically featured between one and five entrances, most commonly two to three, interconnected by a thick silk tube (Figure 2a). The tube could be obscured within a crevice, under debris or exposed. Entrances branched from the burrow, with the exposed tube forming curved configurations in ‘Y’, ‘U’, or bent structures (Figure 2a). This tube was usually covered in debris, including excavated material, bark, leaves and other detritus (Figure 2a). Surrounding each entrance was a capture zone, which consisted either of a dense silk sheet extending a short distance from the entrance or a dense lattice with small cells (Figure 2a). The lattice radiated outwards in a fan‐like arrangement, with cells irregular in size, shape and distribution, becoming progressively larger and more elongated outwards (Figure 2a). Long sensory triplines extended outward from the lattice in a wide‐angled distribution (Figure 2a). Triplines from different entrances could intersect at the outer limits of their respective sensory ranges (Figure 2a). We found that *H. cerberea* web design varied with the environment, primarily through variation in the structure in which the burrow was built (Figure 2b–d).

**FIGURE 2:**
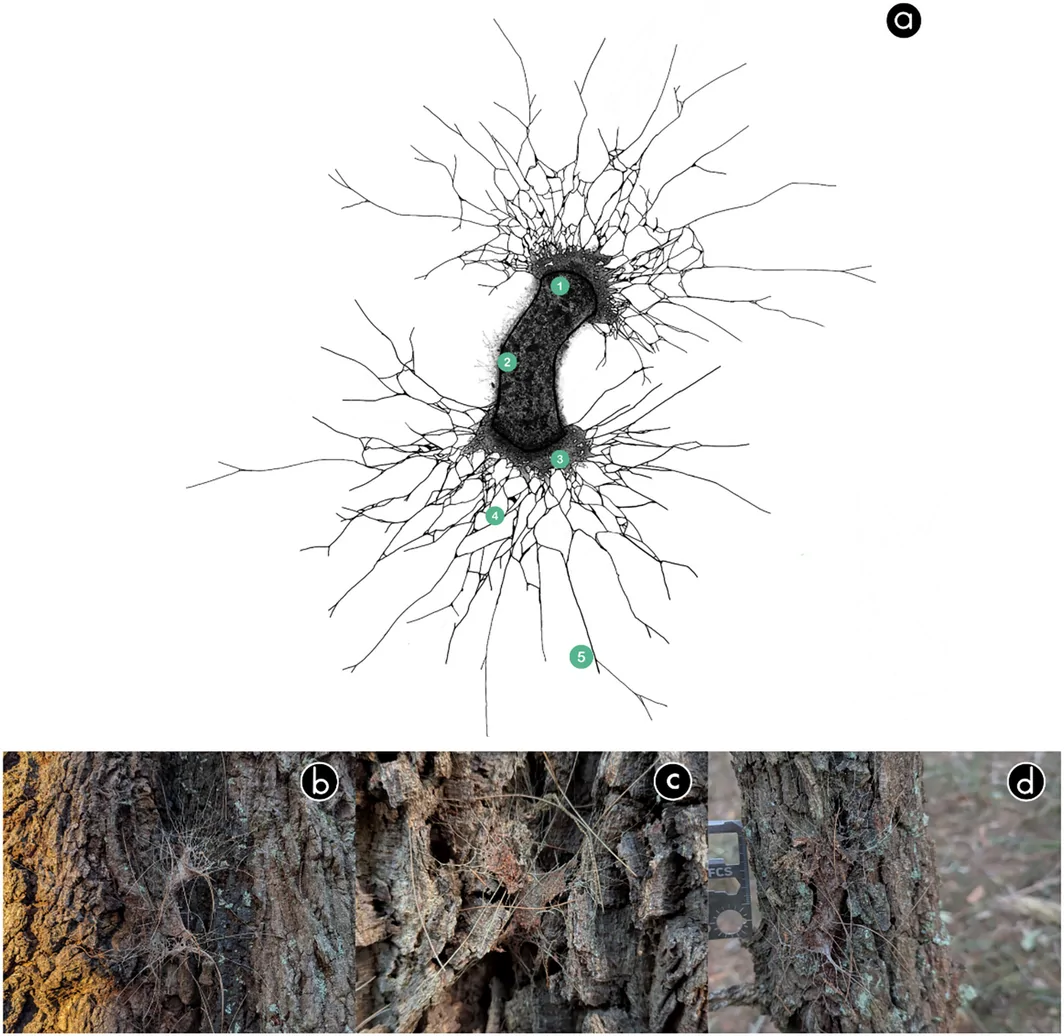
*Hadronyche cerberea* schematic diagram of web architecture (a), with numbered labels indicating key structural features: (1) entrance, (2) silk tube, (3) dense silk lattice, (4) silk lattice and (5) triplines. Photographs (b–d) show examples of *H. cerberea* webs in natural environments, illustrating observed variation. The diagram and photographs do not represent the full range of structural diversity recorded across individuals.

#### 3.1.2 *Atrax robustus* Complex

The *Atrax robustus* complex ranges from Newcastle south through the Illawarra and into the Northern Southern Highlands, with a western distributional limit in the Blue Mountains (Loria et al. 2025). We found individuals only in dry and wet sclerophyll habitats and they were relatively opportunistic in their choice of sites, with burrows in clay embankments, under rocks and logs, at the base of trees or roots and within rotting wood. In several cases, web structure was heavily obscured by debris or surrounding structures, leaving entrances or triplines only partially visible externally.

The stereotypical web design we recorded for the *Atrax* complex was asymmetrical and structurally variable. Webs commonly had multiple entrances (two to six) interconnected by a dense‐mesh silk atrium of varying thickness and shape (Figure 3a), whereas single‐entrance webs generally lacked an extensive atrium and showed minimal extension beyond the burrow (Figure 3b). Where present, the atrium sometimes enclosed a centrally positioned entrance (Figure 3c), but in other cases the entrance occurred at the atrium's edge (Figure 3a). Entrances had concave crescent flaps that were held slightly open even when not in use (Figure 3a). The capture zone comprised a dense lattice of small, irregular cells or a fine silken mesh with no distinct lattice (Figure 3a). This capture structure was often shared among multiple entrances and could extend across the atrium (Figure 3a). Lattice density rarely decreased with distance from the entrance; instead, the capture zone typically terminated into triplines or into small irregular patches of lattice before transitioning into triplines (Figure 3a). Triplines extended outward erratically and varied in direction and length, with some originating from entrance edges and crossing the capture zone; triplines also branched and intersected (Figure 3a).

**FIGURE 3:**
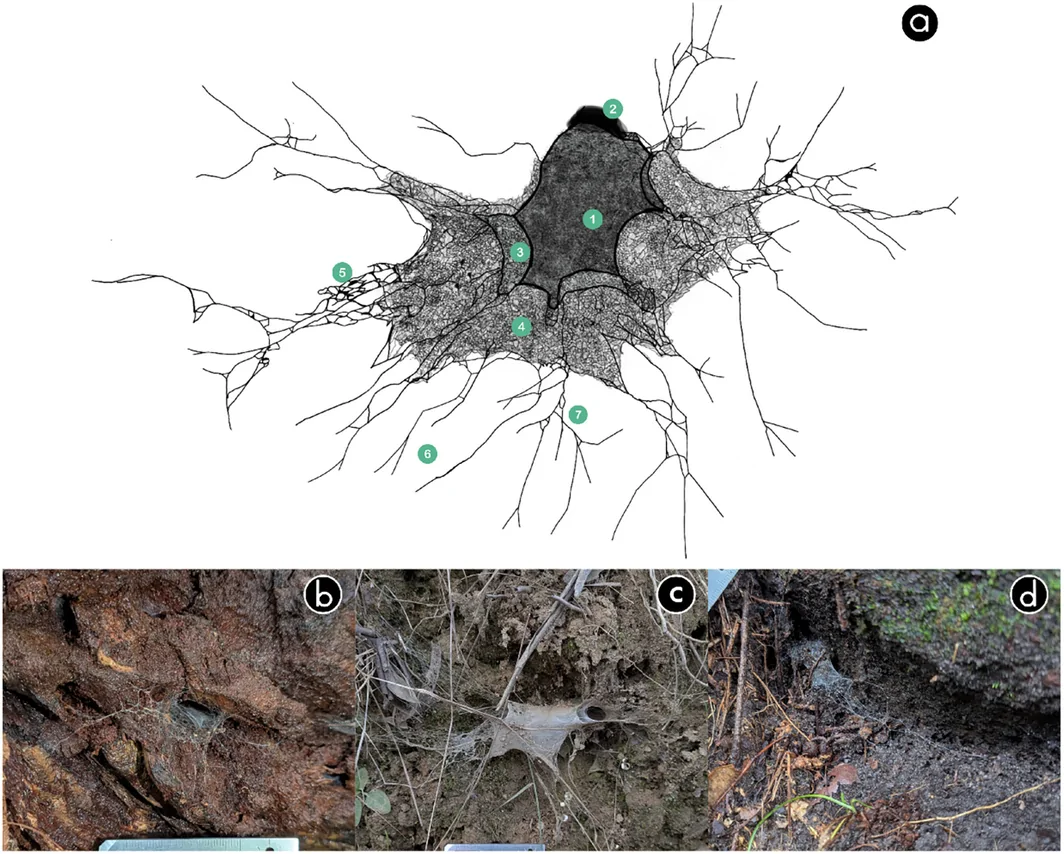
*A. robustus* complex schematic diagram of web architecture (a), with numbered labels indicating key structural features: (1) silk atrium, (2) burrow, (3) web entrance, (4) silk lattice sheet, (5) lattice, (6) triplines and (7) erratic branched triplines. Photographs (b–d) show examples of *A. robustus* complex webs in natural environments, illustrating observed variation. The diagram and photographs do not represent the full range of structural diversity recorded across individuals.

#### 3.1.3 Hadronyche versuta

We recorded *Hadronyche versuta* from the Blue Mountains to the Northern Southern Highlands; its documented range extends into the Illawarra region (Gray 2010). We found *H. versuta* in a variety of substrates, including cracks in sandstone escarpments on cliff edges in dry sclerophyll forest, living tree crevices close to the ground, rotting eucalyptus logs in wet sclerophyll gully forest and soil under roots and rocks in wet sclerophyll near creek lines. *Hadronyche versuta* could occur in dense local populations; in the Blue Mountains, smaller individuals were found within the same log and in sandstone crevices burrows were clustered around larger webs. These smaller individuals built webs of the same overall design (open‐hole entrance with a surrounding lattice and radiating triplines) but at a reduced scale and with a less developed lattice (see also Figure 4b–d).

**FIGURE 4:**
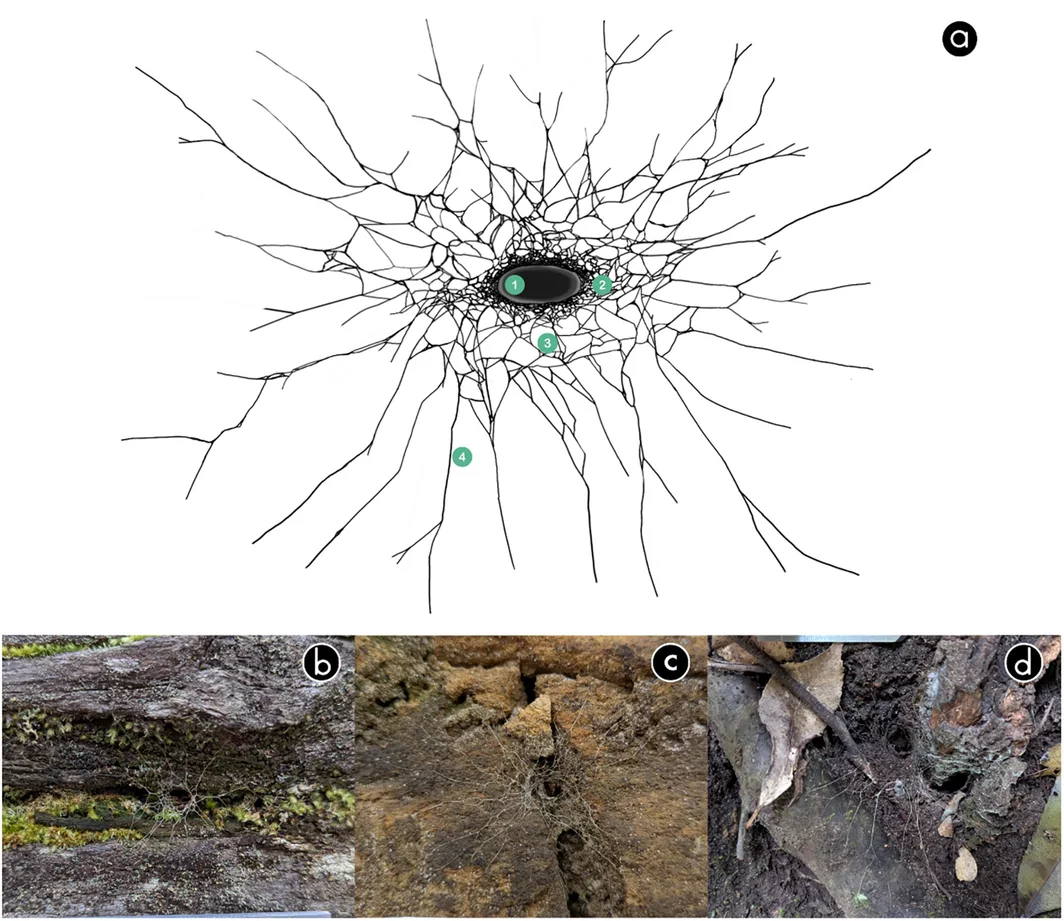
*Hadronyche versuta* schematic diagram of web architecture (a), with numbered labels indicating key structural features: (1) entrance, (2) dense silk lattice, (3) silk lattice zone and (4) triplines. Photographs (b–d) show examples of *H. versuta* webs in natural environments, illustrating observed variation. The diagram and photographs do not represent the full range of structural diversity recorded across individuals.

The typical web design we recorded for *H. versuta* consisted of a relatively symmetrical web with an open‐hole entrance that extended only slightly beyond the burrow (Figure 4a). The silk entrance often sagged slightly, giving an oval outline. Surrounding the entrance was a capture zone consisting of a dense lattice of thick silk with small cells radiating fully around the burrow; in some individuals, the capture structure was instead a thick silk sheet with no obvious lattice (Figure 4a). Where present, lattice density decreased and cell size increased away from the entrance (Figure 4a). From the outer edge of the capture structure, triplines extended further, radiating from regions most in contact with surrounding substrate (Figure 4a). Triplines were most prevalent on the side aligned with the direction the spider emerged from the burrow. Web structure varied with local environment: tripline number and length depended on adjacent substrates around the burrow entrance and smaller individuals showed a less developed lattice with only a few cells surrounding the entrance (Figure 4b–d). We also observed that *H. versuta* sometimes incorporated substrate and burrow debris into the silk lattice surrounding the entrance, although this was not present in every web (Figure 4d).

### 3.2 Statistical Results

#### 3.2.1 Web Area

Mean sensory and capture zone areas varied among the three species (Figure 5a). The *Atrax robustus* complex had the smallest sensory (5940 ± 9948 mm^2^) and capture (870 ± 1059 mm^2^) areas. *Hadronyche versuta* had slightly larger sensory (7705 ± 8434 mm^2^) and capture (983 ± 1032 mm^2^) areas than the *A. robustus* complex. *Hadronyche cerberea* had the largest sensory (13,230 ± 7153 mm^2^) and capture (1436 ± 986 mm^2^) areas. Differences among taxa were most pronounced for the sensory zone, whereas capture zone areas were comparatively similar between *H. versuta* and the *A. robustus* complex.

**FIGURE 5:**
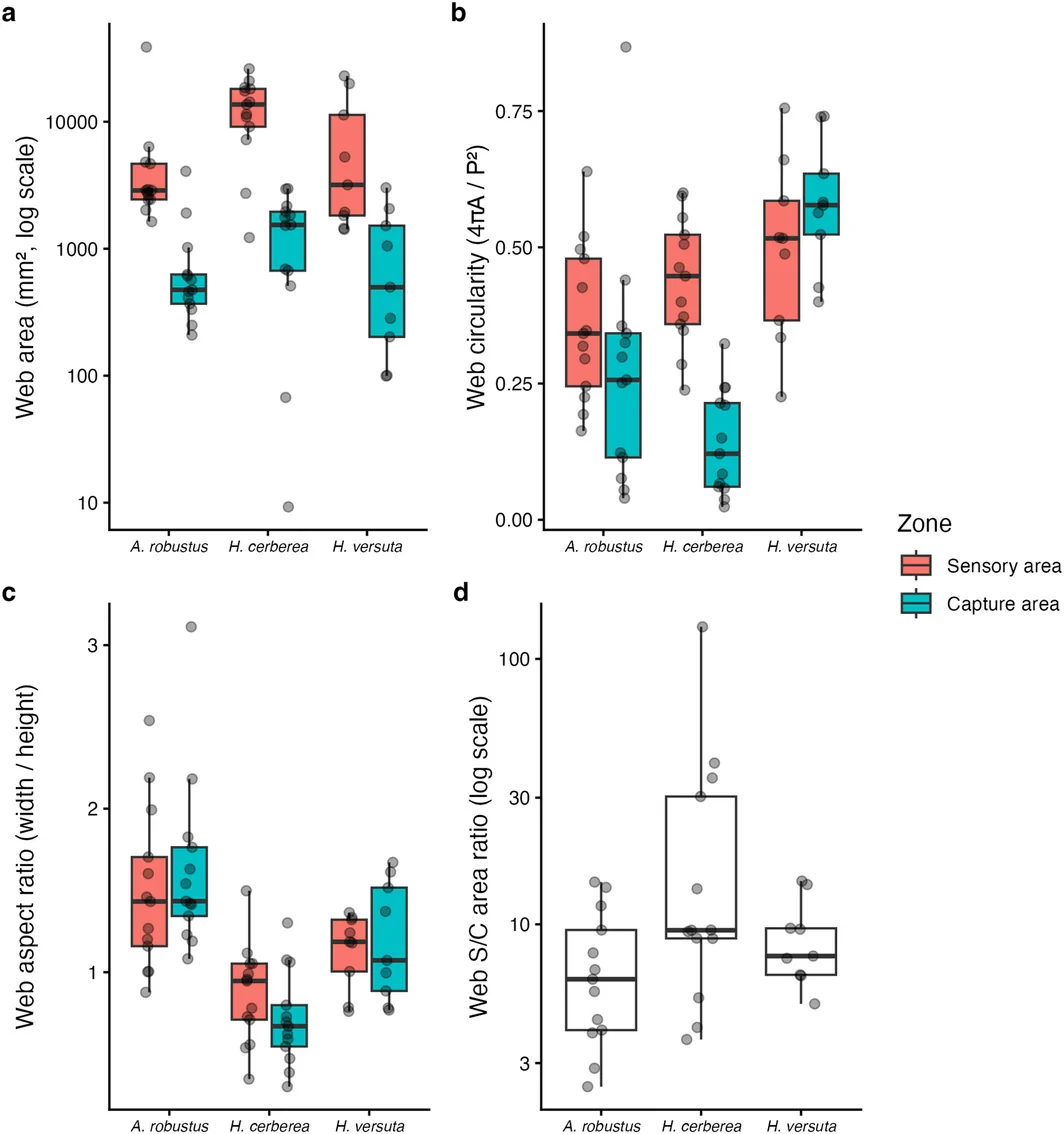
Boxplots comparing web architecture metrics among *Atrax robustus* (*n* = 13), *Hadronyche cerberea* (*n* = 13) and *Hadronyche versuta* (*n* = 9). Panels show (a) web area (mm^2^) (log scale), (b) web circularity, (c) web aspect ratio and (d) sensory‐to‐capture zone (SZ/CZ) area ratio (log scale). Coloured boxes represent sensory (red) and capture (blue) zones. Boxes show interquartile range, horizontal lines indicate medians and whiskers extend to 1.5× the interquartile range. Black points indicate individual webs.

#### 3.2.2 Web Circularity

On average, *H. versuta* had the highest mean circularity values, with similar circularity in the sensory (0.49 ± 0.17) and capture (0.58 ± 0.12) zones (Figure 5b). Unlike the other taxa, *H. versuta* had slightly greater circularity in the capture zone than the sensory zone. *Hadronyche cerberea* showed a markedly more circular sensory zone (0.44 ± 0.12) than capture zone (0.14 ± 0.10), producing the lowest capture circularity values and the largest difference between zones. The *A. robustus* complex had low circularity in both the sensory (0.36 ± 0.14) and capture (0.27 ± 0.22) zones, indicating irregular outlines across web regions (Figure 5b).

#### 3.2.3 Web Aspect Ratio

For the *A. robustus* complex, both sensory (1.49 ± 0.50) and capture (1.63 ± 0.53) zones were wider than they were high (Figure 5c). *Hadronyche cerberea* showed the opposite pattern, with higher sensory (0.87 ± 0.30) and capture (0.71 ± 0.29) zones and greater variation in capture‐zone aspect ratio. *Hadronyche versuta* showed the most uniform proportions, with similar sensory (1.13 ± 0.23) and capture (1.19 ± 0.36) aspect ratios (Figure 5c).

#### 3.2.4 Sensory Zone‐To‐Capture Zone Area Ratio

The *A. robustus* complex showed the most similar sensory‐to‐capture zone area ratio (7.18 ± 4.04; Figure 5d). *Hadronyche versuta* had a similar ratio (8.98 ± 3.38), with lower variability. In contrast, *H. cerberea* showed a substantially larger and more variable ratio (23.94 ± 34.75) than the other taxa.

#### 3.2.5 Principal Component Analysis

Principal component analysis (PCA) indicated that the first two components explained 68.6% of total variation among taxa (Figure 6). PC1 accounted for 37.8% of the variation and was primarily associated with the sensory‐to‐capture zone area ratio. PC2 explained a further 30.8% of the variation and was most strongly influenced by web area and aspect ratio, reflecting differences in overall web size and shape.

**FIGURE 6:**
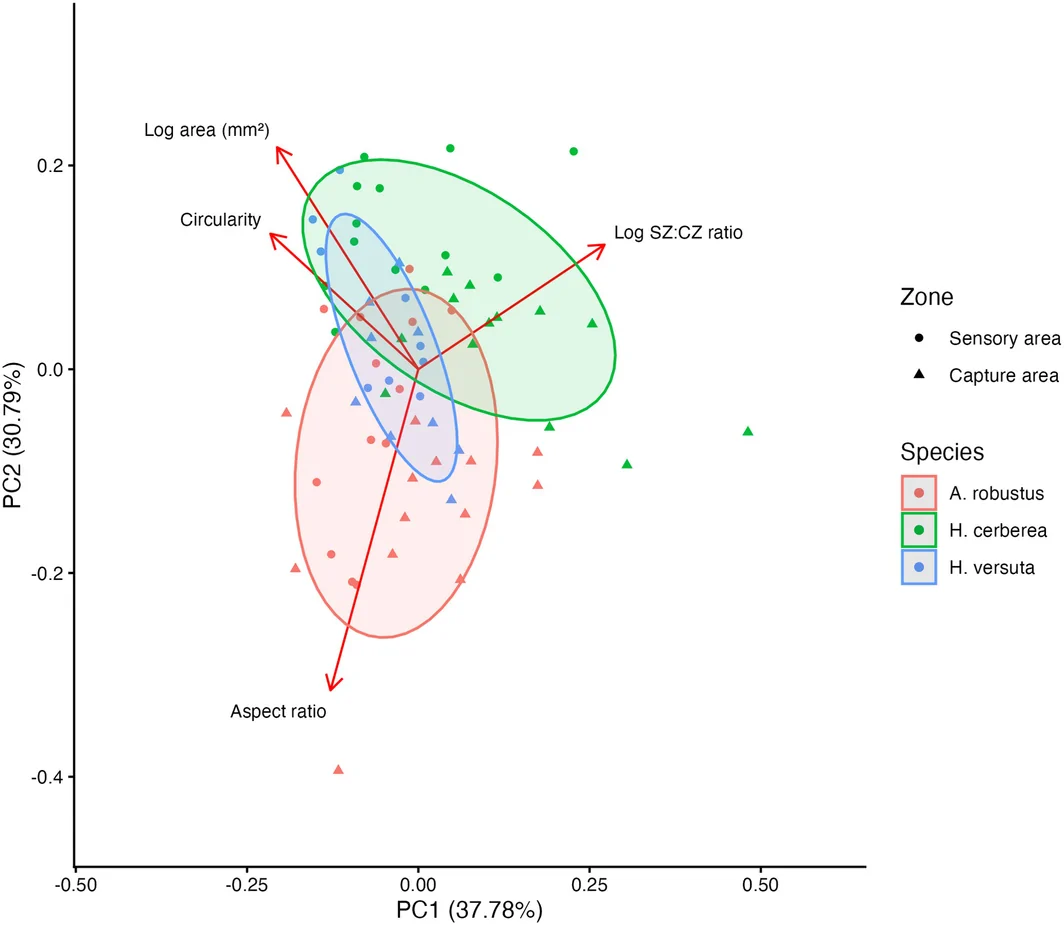
Principal component analysis ordination illustrating inter‐ and intraspecific variation in web architecture among *Atrax robustus* , *Hadronyche cerberea* and *Hadronyche versuta* . PC1 reflects overall functional investment and PC2 reflects overall web shape. Points represent individual webs by zone (circles = sensory, triangles = capture). Red vectors show variable loadings and coloured ellipses indicate 1 standard deviation for each species.

Taxa separated along these axes. The *A. robustus* complex clustered lower on PC2 and between approximately −0.2 and 0.0 on PC1, indicating smaller, more elongated webs, with greater spread along PC2. *Hadronyche cerberea* clustered highest on both PC2 and PC1, with greater spread along PC1, consistent with larger webs and a larger sensory zone relative to the capture zone. *Hadronyche versuta* formed the tightest cluster, with a narrower range on PC1 than PC2, consistent with moderately round webs and comparatively low variation.

PERMANOVA supported these patterns, indicating significant differences among web zones (df = 1, Sum of squares = 39.493, *R* ^2^ = 0.143, *F* = 14.99, *p* < 0.001) and taxa (df = 2, Sum of squares = 62.729, *R* ^2^ = 0.22728, *F* = 11.912, *p* < 0.001). Pairwise comparisons among taxa indicated significant differences between the *A. robustus* complex, *H. cerberea* and *H. versuta* (all pairs *p* < 0.05).

## 4 Discussion

The architecture of mygalomorph spider webs remains poorly characterised despite its potential to reveal key functional and ecological adaptations. In this study, we investigated qualitative and quantitative variation in web architecture across three Atracidae species: *Hadronyche cerberea* , *H. versuta* and the *Atrax robustus* complex. Our results revealed statistically significant interspecific differences across several web metrics, supported by the structural patterns described in our revised qualitative observations. Intraspecific variation was also evident, with consistent trends within species. Below, we interpret these patterns within the limited literature on mygalomorph web architecture, drawing on broader principles of web function, predatory behaviour and environmental constraint to propose plausible functional and evolutionary drivers. We note at the outset that these functional interpretations are inferential rather than demonstrated, as we did not observe prey capture, quantify prey availability or manipulate web structure and the architecture–function links proposed below should therefore be read as hypotheses for future testing.

### 4.1 Interspecific Web Variation

The three species exhibited clear morphometric differences. PCA identified the main axes of variation, with PC1 representing relative functional investment in web components and PC2 reflecting overall web shape (Figure 6). *Hadronyche cerberea* was the most readily distinguishable species, characterised by a markedly greater sensory‐to‐capture zone ratio, larger sensory zone area and lower aspect ratio. Its architecture reflects substantial investment in its sensory zone, defined by long, conspicuous triplines fanning outward from the entrance, while a comparatively smaller, non‐circular capture zone surrounds each entrance of the tube‐like silk structure.

In contrast, the *A. robustus* complex constructs webs with a smaller sensory‐to‐capture zone ratio and greater aspect ratio, indicating proportionally greater investment in the capture zone. Its webs are the most irregular in design, with strong variation in shape and structure, though generally wider, with capture and sensory zones of similar shape—quantitatively affirming previous descriptions of its irregularity (Main 1964). *Hadronyche versuta* occupies an intermediate position in functional investment, though it is perhaps most similar to *A. robustus* in this regard. Its webs are typified by high circularity and regular shape in both zones and it shows the lowest overall variation, indicating a more uniform architecture.

There is limited prior research on web structure in our focal species or near relatives, which constrains direct comparison. Nonetheless, our quantitative findings align with our qualitative descriptions, most notably regarding overall web shape and the structure of triplines and denser silk lattices or sheets that define the sensory and capture zones, respectively. For the same reason, we have deliberately refrained from attempting a broader comparative treatment of web design across Mygalomorphae: quantitatively comparable descriptors are not yet available for most genera and a phylogenetically explicit account of web evolution must await such data.

### 4.2 Intraspecific Web Variation

All three species exhibited measurable intraspecific variation, though along different axes. *Hadronyche cerberea* and *A. robustus* were the most variable, but in opposing ways: *H. cerberea* varied primarily in functional investment (PC1), with individuals differing in capture zone allocation while maintaining a consistent overall shape, largely reflecting differences in silk tube configuration and entrance number. In contrast, *A. robustus* varied most in web shape (PC2), showing plasticity in overall form while consistently emphasising the capture zone. *Hadronyche versuta* showed the least variation overall.

Such variation is likely shaped by both microhabitat and ontogeny. Mygalomorphs often adapt web architecture to local substrate and burrow orientation while preserving core sensory and capture functions (Coyle 1986; Eberhard 2020; M'Rabet et al. 2007) and more exposed habitats may drive individuals to extend their webs—a pattern most evident in the arboreal *H. cerberea* , paralleling observations in the New Zealand tunnel‐web *Porrhothele antipodiana* (Coyle 1986; Laing 1973, 1975). Ontogeny also contributes: in both *H. cerberea* and *H. versuta* , smaller and likely more juvenile individuals built simpler, smaller designs, consistent with patterns seen in tarantulas where web complexity increases with maturity (Eberhard 2020; M'Rabet et al. 2007). This pattern was less evident in *A. robustus* , though the largest individuals had the most complex structures.

### 4.3 Functional Interpretation of Web Designs

Atracidae rely primarily on substrate‐borne vibrations for prey detection, with webs functioning as extensions of the spider's sensory range (Buchli 1969; Coyle 1981, 1986; Wilson et al. 2023). Their architecture shows a more distinctive functional division than other web‐building mygalomorphs: triplines extending outward form the sensory zone, while silk sheets and lattices nearest the entrance impede prey movement and form the capture zone (Coyle 1986; Coyle and Ketner 1990; Gray 2010; Laing 1973, 1975). The three species balance these components differently. *Atrax robustus* invests heavily in its capture zone through fine silk sheets but has a reduced sensory range, while both *Hadronyche* species invest in dense lattice capture zones and allocate proportionally more to their sensory zones—a pattern most extreme in *H. cerberea* .

The architecture of *H. cerberea* appears adapted to arboreal conditions, where reduced prey abundance and elevated predation risk from visual predators such as birds may favour extensive sensory coverage over a large capture area (Coyle 1986; Gray 1984, 1988; Laing 1975; Wilson et al. 2023). Its elongated web oriented toward the tree base and canopy, may align with prey movement patterns—a hypothesis worth direct testing—and a smaller capture zone limits exposure by reducing the distance the spider must travel from its camouflaged, reinforced silk tube. *Hadronyche versuta* , by contrast, lacks an obvious defensive structure despite facing comparable terrestrial predators (Gray 1984, 1988). It may instead compensate through earlier threat detection via an extended sensory field, functionally analogous to open‐hole and collar architectures in other mygalomorphs (Coyle 1986). Although the absolute sensory‐zone area of *H. versuta* is intermediate among the three taxa, the term ‘extended’ here refers to the broadly radial distribution of triplines around the entrance, which provides near‐uniform angular coverage; in proportional terms, *H. versuta* still allocates marginally more of its web to the sensory zone than *A. robustus* (mean SZ:CZ = 8.98 vs. 7.18). Both *A. robustus* and *H. cerberea* appear to have increased entrance number and orientation, potentially offsetting the foraging area lost to their respective protective structures (Coyle 1971, 1981, 1986; Wilson et al. 2023).

Together, these patterns illustrate how atracid web architecture mediates trade‐offs between prey detection, capture efficiency and predator avoidance. Although these interpretations remain preliminary, they offer testable hypotheses linking ecological context, microhabitat and silk‐based behavioural adaptations and expanding comparative work across genera will be key to resolving the evolutionary significance of web diversity in this lineage (Coyle 1986; Wilson et al. 2023).

### 4.4 Applied Insights

Within mygalomorphs, burrow architecture can serve as a valuable diagnostic feature. This approach has been applied successfully to distinguish *Euoplos* and *Cryptoforis* species, including sympatric populations, based on burrow traits (Rix et al. 2017; Wilson et al. 2018; Wilson et al. 2020; Wilson et al. 2019). The consistent differences in web architecture identified here provide a similar basis for reliable field identification of *H. versuta* , *H. cerberea* and the *A. robustus* complex. Crucially, this offers a non‐invasive alternative to excavating individuals—a process that poses significant safety risks with Atracidae—and has practical applications for ecological surveys, medical identification, antivenom collection and future research.

More broadly, web architecture is an integrative trait linking behaviour, morphology, microhabitat use and evolutionary history. The sedentary nature, extended lifespans, clustered populations and burrow fidelity of mygalomorphs make them excellent models for long‐term studies of web‐building behaviour and its plasticity under environmental change (M'Rabet et al. 2007; Main 1982; Pérez‐Miles and Perafán 2017; Wilson et al. 2023).

## 5 Conclusion

Atracidae offer a compelling system for investigating the functional significance of silk architecture in mygalomorphs, given their distinctive division of webs into sensory and capture components. Our results—the first quantitative characterisation of web architecture in this family—demonstrate that species‐specific differences are measurable, consistent and interpretable within an ecological framework. Expanding this work to include additional species, particularly *Illawarra* and integrating behavioural assays of prey detection and capture with architectural data would allow the functional hypotheses proposed here to be tested directly. The consistent interspecific differences we identified also support the use of web architecture as a non‐invasive diagnostic tool, with immediate practical applications for field surveys, medical identification and antivenom collection in a group where handling live specimens carries serious risk. More broadly, the quantitative framework presented here is readily applicable across Mygalomorphae, offering a path toward understanding how silk use and web architecture have shaped the ecological and evolutionary success of one of the oldest lineages of spiders.

## Author Contributions

**Thomas E. White:** conceptualization (equal), data curation (equal), formal analysis (equal), funding acquisition (lead), project administration (lead), resources (lead), visualization (equal), writing – review and editing (lead). **Noah Brown:** conceptualization (equal), data curation (equal), formal analysis (equal), investigation (equal), methodology (equal), visualization (equal), writing – original draft (equal).

## Funding

The authors have nothing to report.

## Conflicts of Interest

The authors declare no conflicts of interest.

## Supporting information


**Data S1:** Supporting Information.

**Figure S1:** Map showing collection sites across Greater Sydney, the Central Coast and the Northern Southern Highlands.

## Data Availability

Data and code are openly archived via Zenodo (https://doi.org/10.5281/zenodo.23149320).
