# Supplementary material for: Inter‐ and Intraspecific Variation in Web Architecture and Its Significance in Australian Funnel‐Web Spiders (Atracidae)
Source: Ecol Evol. 2026 Oct 14;16(10):e74473. doi: 10.1002/ece3.74473 (PMC13668323; doi:10.1002/ece3.74473)
Supplement: Supplementary file 1 [file ECE3-16-e74473-s001.pdf]

Supplementary material for: Brown & White (2026) Inter- and Intraspecific Variation in Web Architecture and its Significance in Australian Funnel-Web Spiders (Atracidae).

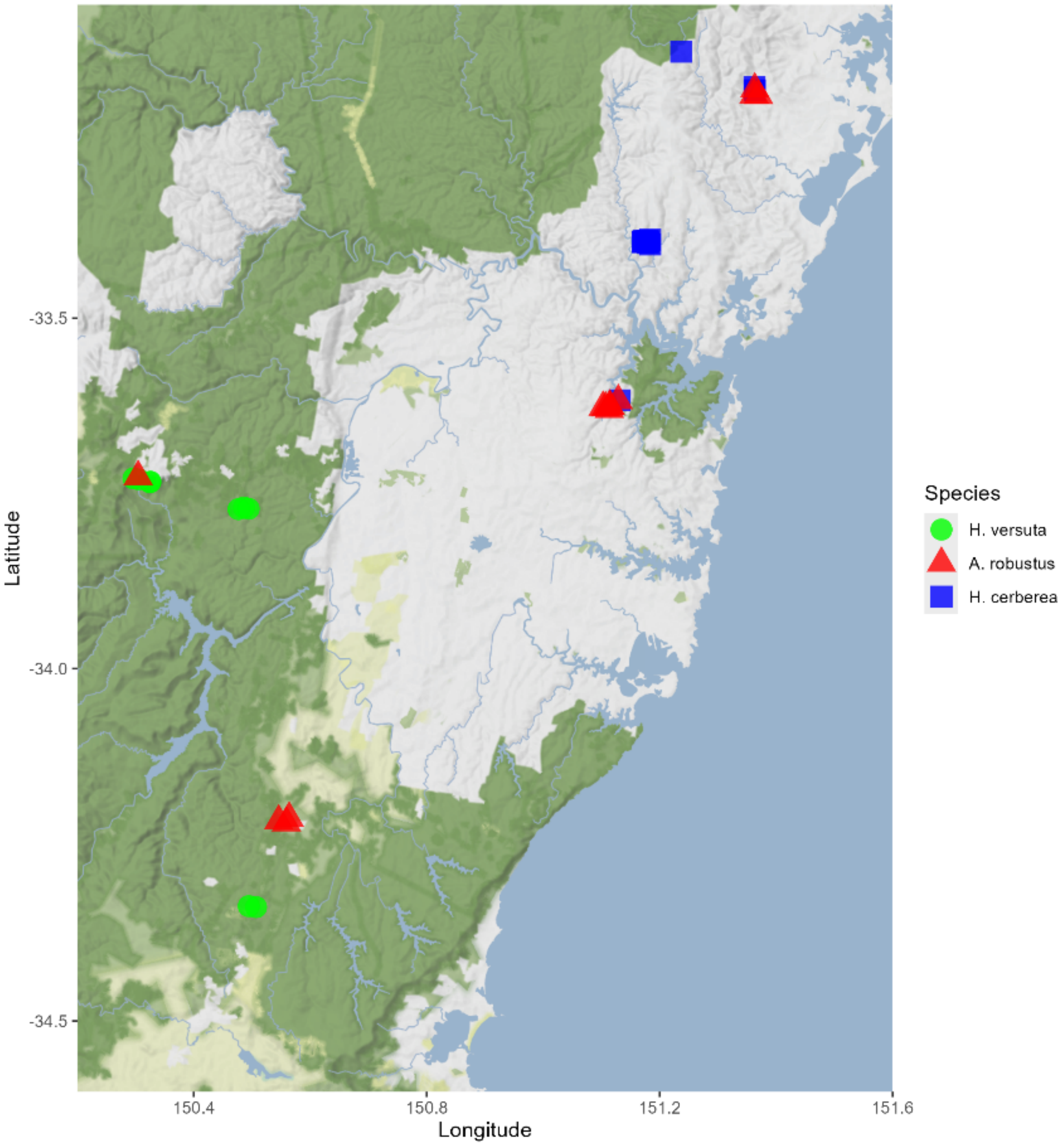

Figure S1: Map showing collection sites across Greater Sydney, the Central Coast, and the Northern Southern Highlands. Symbols indicate the geographic distribution of the recorded species: *Hadronyche versuta* (green circles), *Atrax robustus* (red triangles), and *Hadronyche cerberea* (blue squares).
